# Study on the stability of antipsychotic drugs in clinical samples within serum collection tubes with and without separating gel

**DOI:** 10.3389/fpsyt.2026.1849104

**Published:** 2026-07-17

**Authors:** Wenjuan Dong, Xin Cheng, Zhi Chu, Haifeng Zhang, Jing He, Xue Zhang

**Affiliations:** 1 Department of Clinical Laboratory, Beijing Xicheng District Pingan Hospital, Beijing, China; 2 Department of Psychiatry, Beijing Xicheng District Pingan Hospital, Beijing, China; 3 Department of Clinical Laboratory, Beijing Anding Hospital, Capital Medical University, Beijing, China; 4 Department of Hematology, Fuxing Hospital, Capital Medical University, Beijing, China

**Keywords:** antipsychotic agents, blood specimen collection, pre-analytic phase, serum, specimen handling, therapeutic drug monitoring

## Abstract

**Background:**

The study evaluated loss due to adsorption of selected antipsychotic drugs in serum samples collected in blood collection tubes with and without separation gel using real clinical specimens.

**Methods:**

Clinical serum specimens (n = 30 per drug) were obtained from paired blood collection tubes with and without separation gel. After clotting for approximately 2 h at room temperature, samples were centrifuged, and Day 0 drug concentrations were measured by LC–MS/MS within 2 h after centrifugation. To distinguish intrinsic analyte instability from separation-gel-associated loss, serum aliquots from both gel and non-gel tubes were stored in the dark in a monitored refrigerator set at 4°C, with recorded temperatures ranging from 3.6°C to 4.8°C, and reanalyzed on Days 2 and 7.

**Results:**

On Day 0, the concentrations of clozapine and aripiprazole were significantly lower in separation-gel tubes than in non-gel tubes, with mean relative reductions of 7.86% and 5.02%, respectively, although both remained within the predefined ±15% acceptance criterion. In non-gel tubes, clozapine remained stable during refrigerated storage, with residual concentrations of 99.1% on Day 2 and 98.3% on Day 7 relative to Day 0. In contrast, clozapine in gel tubes decreased to 95.0% on Day 2 and 89.6% on Day 7. The gel versus non-gel difference was significant on Day 7, with a mean additional loss of 8.7% in gel tubes compared with non-gel tubes (p < 0.001). Aripiprazole showed a lower concentration in gel tubes on Day 0 but no further time-dependent loss in either tube type during storage. No relevant loss was observed for the remaining antipsychotic drugs and metabolites.

**Conclusion:**

Under the tested conditions, most analytes showed no clinically relevant concentration loss during 7 days of refrigerated storage. Clozapine showed a measurable decrease in separation-gel tubes but not in non-gel tubes, suggesting an additional gel-associated contribution that may affect TDM interpretation near clinical decision thresholds. Aripiprazole showed an early tube-related difference without further storage-dependent loss. These findings should be interpreted within the evaluated tube brand, gel formulation, storage condition, and concentration ranges.

## 1 Introduction

Therapeutic drug monitoring (TDM) relies on accurate measurement of drug concentrations in biological samples to guide clinical decisions (1, 2). For antipsychotics with narrow therapeutic windows, such as clozapine, even small pre-analytical variations can introduce imprecision and potentially lead to clinically meaningful misinterpretation (3, 4).

During the pre-analytical phase, measured drug concentrations may be affected by blood collection materials, sample handling, and storage conditions (5). A recognized source of pre-analytical bias is adsorption-related drug loss associated with serum separator gel tubes. Although separation gels are designed to physically separate serum from cellular components, several studies have shown that therapeutic drugs can be adsorbed by barrier gels in a time-dependent and compound-dependent manner, resulting in falsely low measured concentrations – particularly for lipophilic drugs (5, 6).

Pre-analytical errors related to blood collection tubes and separator gels were comprehensively reviewed by Peck, Palmer, and Dasgupta, who identified gel-associated adsorption as a persistent and underrecognized source of error in TDM (6). However, previous investigations have often used spiked serum, spiked plasma, or forensic/toxicology samples rather than paired authentic clinical TDM specimens. Such designs may not fully reflect routine clinical conditions, where endogenous proteins, metabolites, protein-binding status, and patient-specific factors can influence drug–gel interactions (7-8).

Thus, uncertainty remains regarding the magnitude and time course of pre-analytical concentration changes in authentic clinical serum samples. Moreover, laboratory quality standards such as ISO 15189 require clinical laboratories to identify, verify, and control pre-analytical factors that may influence test results (9). Therefore, evidence derived from authentic patient specimens is needed to clarify the practical impact of separator-gel-associated drug loss under routine laboratory conditions.

The present study had two specific aims: first, to compare Day 0 serum concentrations of antipsychotic drugs collected in tubes with and without separator gel, thereby evaluating the potential adhesive or adsorption-related effect of the gel; and second, to assess short-term refrigerated stability on Days 2 and 7 in gel and non-gel serum aliquots, using non-gel tubes as reference controls for intrinsic analyte stability.

## 2 Materials and methods

### 2.1 LC–MS/MS instrumentation, reagents, and standards

All serum samples were analyzed using a high-performance liquid chromatography–tandem mass spectrometer (AB SCIEX Triple Quad 4500 MD, AB Sciex, Framingham, MA, USA) equipped with an electrospray ionization (ESI) source operating in positive mode. LC–MS/MS analysis was performed using an AB SCIEX Triple Quad 4500 MD (Framingham, MA, USA) with electrospray ionization in positive mode. Chromatographic separation was performed on a C18 column (100 × 2.1 mm, 1.7 μm; Waters, Milford, MA, USA) under gradient elution with 0.1% formic acid in LC-MS-grade water and LC-MS-grade methanol. Standards and stable isotope-labeled internal standards were obtained from certified suppliers (purity ≥96–99%). LC-MS-grade water was produced by a Milli-Q system (Millipore, Burlington, MA, USA). The gradient ran from 10% to 90% B over 6 minutes at a flow rate of 0.3 mL/min, column temperature 40°C, and injection volume 5 µL.

Detection was performed in multiple-reaction-monitoring (MRM) mode using transitions specific for each analyte and corresponding stable isotope–labeled internal standard (Cayman Chemical, Ann Arbor, MI, USA). Quantification was achieved by isotope dilution calibration. Calibration curves and quality-control samples were prepared in drug-free human serum obtained from certified commercial suppliers (Biological Specialty, Colmar, France). Reference standards for all analytes and metabolites were obtained from certified suppliers (≥96–99% purity), and stable isotope-labeled internal standards had ≥98% purity. Drug-free human serum for calibration and quality control was obtained from Biological Specialty, Colmar, France.

Acetonitrile (LC-MS grade, Merck, Darmstadt, Germany), methanol (LC-MS grade, Merck, Darmstadt, Germany), and 0.1% formic acid (Sigma-Aldrich, St. Louis, MO, USA) were used for sample preparation and mobile phases. LC-MS-grade water was produced by a Milli-Q system (Millipore, Burlington, MA, USA).

Reference standards for aripiprazole, olanzapine, quetiapine fumarate, risperidone, paliperidone, clozapine, dehydroaripiprazole, dealkylquetiapine, and norclozapine were obtained from Sigma-Aldrich (St. Louis, MO, USA) or Merck (Darmstadt, Germany), with stated purities of ≥96% for dealkylquetiapine, ≥98% for risperidone, paliperidone, dehydroaripiprazole, and norclozapine, and ≥99% for aripiprazole, olanzapine, quetiapine fumarate, and clozapine. Stable isotope-labeled internal standards (Cayman Chemical, Ann Arbor, MI, USA) were obtained with stated purities of ≥98%.

#### 2.1.1 Blood collection tubes and serum matrices

Two types of blood collection tubes were used in this study: VACUETTE^®^ CAT Serum Separator Clot Activator Tubes (with gel) and VACUETTE^®^ CAT Serum Clot Activator Tubes (without gel) (Greiner Bio-One GmbH, Kremsmünster, Austria). These tubes represent two variants of the same brand, differing only by the presence or absence of a separation gel barrier.

### 2.2 Methods

#### 2.2.1 Study setting, design, patients, and sample collection

This study was conducted in the Clinical Laboratory Department of Beijing Xicheng District Pingan Hospital. The work was designed as an analytical study using residual clinical serum specimens obtained during routine therapeutic drug monitoring (TDM) for antipsychotic medications.

Adult psychiatric patients receiving one of six antipsychotic drugs (aripiprazole, olanzapine, quetiapine, risperidone, paliperidone, or clozapine) were recruited consecutively through routine TDM requests. Inclusion criteria were: (1) age ≥ 18 years, (2) treatment with a single monitored antipsychotic from the study list, and (3) sufficient remaining serum volume after completion of routine tests. Exclusion criteria included: (1) visibly hemolyzed samples, which were excluded because hemolysis can interfere with matrix effects, reduce quantitative accuracy, and compromise sample integrity; (2) insufficient serum volume; (3) missing or unclear medication information; and (4) complex multi-drug regimens that could not be clearly attributed to a single index antipsychotic. The exclusion of hemolyzed samples ensures reliable LC–MS/MS quantification and minimizes analytical bias.

For each index antipsychotic, 30 paired serum specimens were included (n = 30 per analyte), resulting in 180 paired specimens collected from 180 independent patients. Each patient contributed only one paired sample corresponding to the antipsychotic they were receiving; therefore, no patient contributed to multiple drug groups. This clarification ensures that each sample pair represents an independent observation for the respective analyte.

Blood collection was performed by trained phlebotomists using standardized hospital procedures. For each patient, paired VACUETTE^®^ tubes (one with gel, one without gel) were drawn at the same venipuncture to ensure comparable pre-analytical conditions for both tube types.

#### 2.2.2 Pre-analytical handling, serum aliquoting, and storage conditions

All blood samples were allowed to clot for approximately 2 h at room temperature before centrifugation. This clotting interval was applied consistently to both gel and non-gel tubes.

After clotting, samples were centrifuged at 3500 × g for 5 min in accordance with the manufacturer’s instructions. Serum was separated immediately after centrifugation. Day 0 measurements for both gel and non-gel tubes were completed within 2 h after centrifugation, not within 2 h after venipuncture.

For longitudinal assessment, serum aliquots were prepared from each gel and non-gel tube immediately after Day 0 processing. Blood samples were collected in VACUETTE^®^ CAT tubes (with and without gel, Greiner Bio-One GmbH, Kremsmünster, Austria) and allowed to clot for ~2 h at room temperature. After centrifugation (3500 × g, 5 min), serum was aliquoted and stored in polypropylene tubes in the dark at 4°C (3.6–4.8°C). Day 0 analysis was performed within 2 h after centrifugation; aliquots were reanalyzed on Days 2 and 7. This design allowed comparison of storage-related changes in gel tubes with parallel non-gel controls, thereby distinguishing intrinsic short-term analyte instability from gel-associated concentration loss.

#### 2.2.3 Sample extraction and LC–MS/MS analysis

Sample preparation was performed using a protein-precipitation protocol compatible with Qiagen extraction systems (Qiagen, Hilden, Germany). In brief, 100 µL of serum was mixed with an organic precipitant containing isotope-labelled internal standards, vortexed, and centrifuged. The supernatant was transferred to 96-well plates and analyzed by LC–MS/MS.

Detection was performed using electrospray ionization in multiple-reaction-monitoring mode.

The analytical method was validated in accordance with the China Pharmacopoeia guidance for quantitative bioanalysis and international bioanalytical method validation recommendations (10, 11). Calibration curves were linear over the clinically relevant concentration ranges for all analytes. Four quality control (QC) levels were used, including the lower limit of quantification (LLOQ), low QC, medium QC, and high QC. Intra-day precision ranged from 2.8% to 7.6%, and inter-day precision ranged from 3.5% to 9.4% across analytes and QC levels. Accuracy, expressed as relative bias, ranged from −6.8% to 7.9%. The LLOQ was 2 ng/mL for risperidone and paliperidone, 5 ng/mL for aripiprazole, olanzapine, quetiapine, dehydroaripiprazole, dealkylquetiapine, and norclozapine, and 10 ng/mL for clozapine. The predefined acceptance criteria were precision within 15% coefficient of variation and accuracy within ±15% of nominal concentrations, except at the LLOQ, where ±20% was accepted. A summary of the analytical measurement ranges, LLOQs, precision, and accuracy is provided in **Table 1**.

**Table 1:** LC–MS/MS method performance for antipsychotic drugs and metabolites.

Analyte	Calibration range (ng/mL)	LLOQ (ng/mL)	Intra-day precision CV (%)	Inter-day precision CV (%)	Accuracy/bias (%)
Aripiprazole	5–1000	5	3.1–6.8	4.2–8.7	−5.4–6.2
Dehydroaripiprazole	5–1000	5	3.4–7.1	4.5–8.9	−6.1–6.8
Olanzapine	5–500	5	2.8–6.5	3.8–8.2	−4.9–5.7
Quetiapine	5–1000	5	3.6–7.4	4.7–9.1	−6.3–7.2
Dealkylquetiapine	5–1000	5	3.8–7.6	4.9–9.4	−6.8–7.9
Risperidone	2–200	2	3.2–6.9	4.1–8.5	−5.7–6.4
Paliperidone	2–200	2	3.0–6.7	3.9–8.3	−5.2–6.1
Clozapine	10–2000	10	3.5–7.2	4.6–9.0	−6.5–7.4
Norclozapine	5–1000	5	3.3–7.0	4.3–8.8	−5.9–6.6

Values represent the observed ranges across four QC levels, including LLOQ, low QC, medium QC, and high QC. Precision was expressed as coefficient of variation. Accuracy was expressed as relative bias from nominal concentrations. Acceptance criteria were based on China Pharmacopoeia guidance and international bioanalytical method validation recommendations: precision within 15% and accuracy within ±15%, except at the LLOQ, where ±20% was accepted (10, 11).

#### 2.2.4 Stability study design: Day 0, Day 2, Day 7

On Day 0, serum concentrations of each drug and metabolite were measured in paired gel and non-gel tubes. For each tube type, residual concentration (%) was calculated as the concentration measured on Day 2 or Day 7 divided by the corresponding Day 0 concentration from the same tube type. In addition, gel-to-non-gel ratios were calculated at each time point to estimate the tube-related effect after correction for intrinsic storage stability.

Non-gel tubes were included at all storage time points as reference controls for intrinsic analyte stability under the same refrigerated conditions. For graphical presentation, residual concentrations were plotted at Day 0, Day 2, and Day 7 for each analyte without connecting individual sample points.

#### 2.2.5 Ethics statement

This study was approved by the Ethics Committee of Beijing Xicheng District Pingan Hospital (Approval No. 2023-kjxx-01). Because paired serum samples were obtained through an additional blood draw at the same venipuncture beyond routine clinical testing, all participants provided written informed consent prior to sample collection. No separate studies used leftover anonymized specimens; therefore, the consent waiver applied only to residual serum used for calibration and quality-control purposes, not for the primary analytical study.

#### 2.2.6 Statistical analysis

All statistical analyses were performed using SPSS software and Microsoft Excel (version 27.0). Data normality was assessed using the Shapiro–Wilk test for each analyte and time point. Normally distributed paired data were compared using paired two-sided t-tests, whereas non-normally distributed paired data were analyzed using Wilcoxon signed-rank tests. No formal adjustment for multiple comparisons was applied, as analyses were predefined and hypothesis-driven. For each analyte, the following comparisons were performed: (1) gel versus non-gel tubes at Day 0 to evaluate the initial tube effect; (2) Day 2 and Day 7 versus Day 0 within each tube type to evaluate storage stability; (3) gel versus non-gel tubes at Days 2 and 7 to estimate additional gel-associated loss after accounting for intrinsic analyte stability. All p values reported in tables indicate these specific comparisons, as described in the table legends. A p value < 0.05 was considered statistically significant.

## 3 Results

### 3.1 Day 0 concentration ranges and tube-related effects

These values (see **Table 2**) were generally within or near commonly used therapeutic reference ranges for antipsychotic therapeutic drug monitoring (5, 12). For clozapine, the observed concentration range covered both lower and higher clinically relevant concentrations, allowing assessment of whether gel-associated loss could affect samples close to therapeutic decision thresholds.

**Table 2:** Day 0 comparison of serum drug concentrations between separation-gel and non-gel tubes.

Analyte	Absolute day 0 concentration in non-gel tubes, median (IQR), ng/mL	Observed range, ng/mL	Gel/non-gel ratio (%), mean ± SD	Mean relative difference (%)	95% CI of relative difference (%)	p value	AA
Aripiprazole	286 (196–412)	84–782	94.98 ± 5.69	−5.02	−7.15 to −2.89	5.42 × 10^-6^	AA1
Olanzapine	28 (18–42)	7–86	99.93 ± 5.18	−0.07	−2.00 to 1.86	0.07	AA2
Quetiapine	218 (126–386)	42–812	99.73 ± 4.83	−0.27	−2.07 to 1.53	0.27	AA2
Risperidone	18 (11–28)	4–62	99.42 ± 4.39	−0.58	−2.22 to 1.06	0.58	AA2
Paliperidone	34 (22–48)	8–96	99.51 ± 4.66	−0.49	−2.23 to 1.25	0.49	AA2
Clozapine	412 (286–568)	128–924	92.14 ± 5.32	−7.86	−9.85 to −5.87	2.70 × 10^-7^	AA3
Dehydroaripiprazole	68 (42–104)	18–216	99.42 ± 4.99	−0.58	−2.44 to 1.28	0.58	AA2
Dealkylquetiapine	96 (54–158)	18–326	99.28 ± 3.85	−0.72	−2.16 to 0.72	0.72	AA2
Norclozapine	236 (158–348)	64–702	99.90 ± 5.05	−0.10	−1.99 to 1.79	0.1	AA2

• Values are expressed as median (IQR), observed range, or mean ± SD, as appropriate.

• Absolute concentrations were measured in non-gel tubes on Day 0. The gel/non-gel ratio was calculated as the concentration measured in the separation-gel tube divided by the concentration measured in the paired non-gel tube on Day 0.

• Mean relative difference was calculated as gel/non-gel ratio minus 100%.

• P values reflect paired Day 0 comparisons between gel and non-gel tubes using paired t-tests for normally distributed data or Wilcoxon signed-rank tests for non-normally distributed data.

• AA (Acceptance Assessment) abbreviations:

• AA1: Within ±15%, statistically significant.

• AA2: Within ±15%.

• AA3: Within ±15%, but statistically significant and clinically relevant.

**Figure 1:**
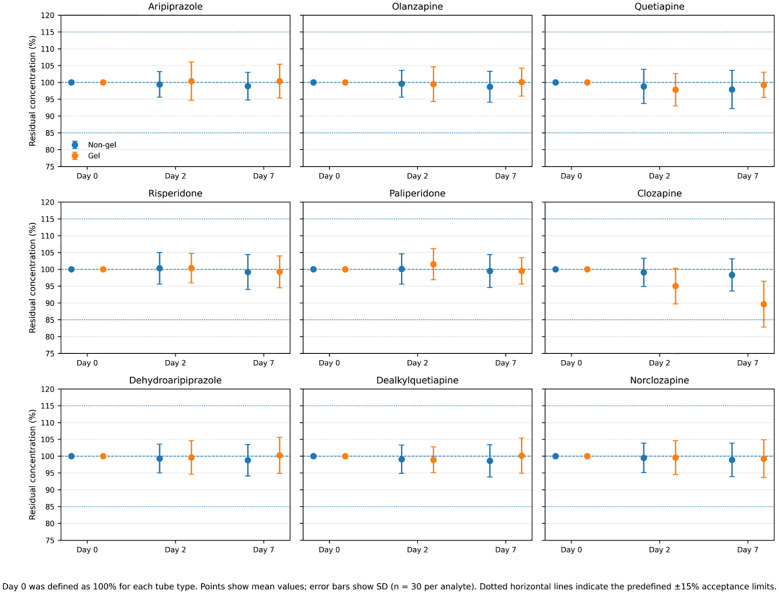
Residual serum concentrations of antipsychotic drugs in gel and non-gel tubes during refrigerated storage at 4 °C. Residual concentrations were calculated relative to the Day 0 concentration of the same tube type, with Day 0 defined as 100%. Each panel represents one analyte. Data are shown for Day 0, Day 2, and Day 7 using boxplots with individual sample points; individual points are not connected. Serum aliquots were stored in the dark in a monitored refrigerator set at 4 °C, with recorded temperatures ranging from 3.6 °C to 4.8 °C. Clozapine remained stable in non-gel tubes but decreased in separation-gel tubes during storage, with residual concentrations of 95.01% on Day 2 and 89.64% on Day 7. Other analytes showed no clinically relevant storage-dependent loss in either tube type. Data represent n = 30 per analyte.

Day 0 tube-related effects, including absolute concentration ranges, gel/non-gel ratios, relative differences, 95% confidence intervals, p values, and acceptance assessment, are summarized in **Table 2**. Storage stability results are summarized in **Table 3** and illustrated in **Figure 1**.

**Table 3:** Storage stability of serum drug concentrations in gel and non-gel tubes during refrigerated storage at 4 °C.

Analyte	Tube type	Day 2/ day 0 (%), mean ± SD	p value	Day 7/ day 0 (%), mean ± SD	p value	Acceptance assessment
Aripiprazole	Non-gel	99.40 ± 3.80	0.39	98.90 ± 4.10	0.18	Within ±15%
Aripiprazole	Gel	100.38 ± 5.69	0.71	100.38 ± 5.04	0.68	Within ±15%
Olanzapine	Non-gel	99.60 ± 4.00	0.58	98.70 ± 4.60	0.21	Within ±15%
Olanzapine	Gel	99.47 ± 5.18	0.52	100.09 ± 4.15	0.91	Within ±15%
Quetiapine	Non-gel	98.80 ± 5.10	0.24	97.90 ± 5.70	0.09	Within ±15%
Quetiapine	Gel	97.83 ± 4.83	0.06	99.27 ± 3.77	0.34	Within ±15%
Risperidone	Non-gel	100.30 ± 4.70	0.75	99.20 ± 5.20	0.43	Within ±15%
Risperidone	Gel	100.36 ± 4.39	0.69	99.24 ± 4.74	0.45	Within ±15%
Paliperidone	Non-gel	100.10 ± 4.50	0.89	99.50 ± 4.90	0.62	Within ±15%
Paliperidone	Gel	101.52 ± 4.66	0.08	99.52 ± 3.92	0.53	Within ±15%
Clozapine	Non-gel	99.10 ± 4.20	0.27	98.30 ± 4.80	0.08	Within ±15%
Clozapine	Gel	95.01 ± 5.32	<0.001	89.64 ± 6.81	<0.001	Within ±15%, but statistically significant and clinically relevant
Dehydroaripiprazole	Non-gel	99.30 ± 4.30	0.38	98.80 ± 4.70	0.19	Within ±15%
Dehydroaripiprazole	Gel	99.60 ± 4.99	0.66	100.25 ± 5.37	0.82	Within ±15%
Dealkylquetiapine	Non-gel	99.10 ± 4.20	0.29	98.60 ± 4.80	0.13	Within ±15%
Dealkylquetiapine	Gel	98.93 ± 3.85	0.17	100.15 ± 5.24	0.88	Within ±15%
Norclozapine	Non-gel	99.50 ± 4.40	0.56	98.90 ± 5.00	0.25	Within ±15%
Norclozapine	Gel	99.57 ± 5.05	0.64	99.23 ± 5.62	0.46	Within ±15%

Values are expressed as mean ± SD, n = 30 per analyte. Residual concentrations were calculated relative to the Day 0 concentration of the same tube type, with Day 0 defined as 100%. P values indicate paired comparisons of Day 2 or Day 7 versus Day 0 within the same tube type. Normally distributed data were analyzed using paired two-sided t-tests, whereas non-normally distributed data were analyzed using Wilcoxon signed-rank tests. The predefined acceptance criterion was ±15% relative change from Day 0. Although the mean Day 7 residual concentration of clozapine in gel tubes remained within the ±15% acceptance interval, the decrease was statistically significant and may be clinically relevant for therapeutic drug monitoring, especially near decision thresholds.

### 3.2 Storage stability in gel and non-gel tubes during refrigerated storage

At Day 0, clozapine and aripiprazole showed statistically significant reductions in separation-gel tubes compared with paired non-gel tubes. Clozapine showed a mean gel/non-gel ratio of 92.14% ± 5.32%, corresponding to a mean relative reduction of 7.86% (95% CI, −9.85% to −5.87%; p = 2.70 × 10^-7^). Aripiprazole showed a mean gel/non-gel ratio of 94.98% ± 5.69%, corresponding to a mean relative reduction of 5.02% (95% CI, −7.15% to −2.89%; p = 5.42 × 10^-6^). For olanzapine, quetiapine, risperidone, paliperidone, dehydroaripiprazole, dealkylquetiapine, and norclozapine, mean gel/non-gel ratios ranged from 99.28% to 99.93%, and no statistically significant Day 0 tube-related difference was observed (p = 0.07–0.72). All Day 0 mean relative differences remained within the predefined ±15% acceptance criterion. These results are summarized in **Table 2**.

During refrigerated storage, clozapine remained stable in non-gel tubes, with mean residual concentrations of 99.10% ± 4.20% on Day 2 and 98.30% ± 4.80% on Day 7 relative to the non-gel Day 0 baseline. In contrast, clozapine in separation-gel tubes decreased to 95.01% ± 5.32% on Day 2 and 89.64% ± 6.81% on Day 7 relative to the gel-tube Day 0 baseline; both decreases were statistically significant compared with Day 0 (both p < 0.001). The additional gel-associated difference was most pronounced on Day 7, with an estimated mean between-tube difference of −8.7% (p < 0.001). Aripiprazole showed a Day 0 tube-related reduction but no further storage-dependent decrease, with residual concentrations of 100.38% ± 5.69% on Day 2 and 100.38% ± 5.04% on Day 7 in gel tubes. Across the remaining analytes, Day 7 residual concentrations ranged from 98.60% to 100.25% in non-gel tubes and from 99.23% to 100.15% in gel tubes. Thus, all analytes remained within the predefined ±15% acceptance range, although the decrease observed for clozapine in gel tubes was statistically significant and may be clinically relevant near therapeutic decision thresholds. Storage stability results are summarized in **Table 3**.

The parallel non-gel control data indicated that short-term refrigerated storage alone did not produce relevant concentration loss for clozapine or the other analytes. Therefore, the progressive decrease observed for clozapine in separation-gel tubes was unlikely to be explained by intrinsic analyte instability alone and was more consistent with an additional gel-associated effect. Detailed statistical results for Day 0 tube-type comparisons and storage-time comparisons are provided in **Tables 2**, **3**.

To explore whether concentration loss was more pronounced at lower concentrations, clozapine samples were stratified according to the Day 0 non-gel concentration (data not shown). Samples below 350 ng/mL showed a larger proportional decrease in gel tubes on Day 7 than samples at or above 350 ng/mL. This finding suggests that gel-associated loss may have greater clinical impact when clozapine concentrations are close to or below therapeutic decision thresholds.

## 4 Discussion

This study evaluated tube-related and storage-related changes in selected antipsychotic drug concentrations in serum samples collected under routine clinical conditions using tubes with or without separation gel. By including non-gel tube controls at Days 2 and 7, the revised design distinguished intrinsic short-term analyte instability from additional gel-associated concentration loss. On Day 0, clozapine and aripiprazole showed lower concentrations in gel-containing tubes than in non-gel tubes. Although these differences were statistically significant, they remained within the predefined ±15% acceptance criterion. However, only clozapine exhibited a progressive reduction during refrigerated storage in gel tubes, whereas it remained stable in non-gel tubes over the same period. These findings are particularly relevant for therapeutic drug monitoring (TDM) of clozapine, where measured concentrations guide dose optimization, adherence assessment, efficacy evaluation, and toxicity risk management (9, 13–15).

The present findings should be interpreted alongside previous studies on separator-gel adsorption and antipsychotic drug stability. Dasgupta et al. reported that barrier gels can absorb therapeutic drugs, causing time-dependent reductions in measured serum concentrations, and advised caution when using such tubes for TDM (7, 16). Schrapp et al. emphasized that standard tubes, gel separator tubes, and mechanical separator tubes may not be interchangeable for all drugs (17). Wollmann et al. observed substantial differences in psychoactive drug concentrations after storage for ≥2 days in gel separator tubes compared with standard serum tubes, supporting the view that gel-associated loss is especially relevant for psychoactive and lipophilic compounds (8). Saar et al. evaluated the stability of 30 antipsychotic drugs in stored blood specimens and showed that stability varies by analyte, concentration, matrix, storage temperature, and duration (18). In comparison, our study found progressive clozapine loss in gel tubes, an early Day-0 reduction for aripiprazole without further storage-dependent loss, and no clinically relevant changes for the remaining analytes under the tested conditions. Differences among studies may arise from tube manufacturer, gel polymer formulation, sample type, storage duration and temperature, concentration range, and the use of authentic patient specimens versus spiked matrices.

A key strength of this study is the use of authentic patient serum specimens rather than spiked serum or artificial matrices. Authentic clinical specimens better reflect routine TDM conditions because they preserve patient-specific matrix composition, endogenous proteins, protein-binding status, metabolites, and clinical variability – particularly important for lipophilic, protein-bound antipsychotic drugs. Nevertheless, authentic specimens have limitations: concentration ranges cannot be as tightly controlled as in spiking experiments, and patient-specific factors (dose, sampling time, metabolism, co-medication) may increase variability. Therefore, our results should be viewed as practical observations under routine clinical laboratory conditions rather than as controlled mechanistic adsorption experiments.

The inclusion of non-gel controls at Days 2 and 7 suggests that the progressive decrease in clozapine concentration in gel tubes was not primarily due to intrinsic short-term instability during refrigerated storage. Instead, the greater decrease in gel tubes supports – but does not prove – an additional gel-associated contribution. For aripiprazole, the early reduction observed on Day 0 without further decrease during storage may indicate that any tube-related interaction, if present, occurred rapidly and approached apparent equilibrium before the first measurement. In contrast, the progressive clozapine decrease may reflect continued tube-related loss during refrigerated storage. These analyte-specific differences may be related to physicochemical properties such as lipophilicity, protein binding, pKa-dependent ionization state, and interaction with polymeric gel materials, consistent with prior reports that gel-related drug loss is compound-dependent and associated with drug properties and storage duration (7-8, 16). However, we did not experimentally assess adsorption kinetics, free drug fractions, or direct drug–gel interactions; therefore, mechanistic conclusions cannot be drawn.

For clozapine, which has a relatively narrow therapeutic reference range, pre-analytical concentration loss during storage may affect clinical TDM interpretation, especially when measured concentrations are close to decision thresholds (9, 13, 13). In this study, clozapine remained stable in non-gel tubes but decreased progressively in separation-gel tubes during refrigerated storage, with an additional gel-associated loss of approximately 8.7% by Day 7. This magnitude may be clinically relevant near decision thresholds. Our clozapine concentration range included samples below, within, and above the commonly used therapeutic reference range. Stratified analysis suggested that lower-concentration samples showed a larger proportional decrease after storage in gel tubes. Hence, tube type and storage duration should be considered when interpreting clozapine serum concentrations, particularly when results are near the lower therapeutic threshold. For the other evaluated antipsychotics and metabolites, relative concentrations remained stable across tube types and storage time points, indicating that adsorption-related effects are analyte-specific rather than universal (19-20).

## 5 Limitations

Several limitations should be acknowledged. First, only one tube brand and gel formulation were evaluated; therefore, our findings should not be generalized to all serum separator tubes. Future studies should include tubes from multiple manufacturers and different gel formulations. Second, plasma samples were not included, precluding serum-plasma comparisons. Third, although non-gel tube controls were added at Days 2 and 7 to assess intrinsic short-term stability, an immediate-centrifugation arm was not incorporated. All samples were allowed to clot for approximately 2 h before centrifugation, consistent with local routine workflow. Consequently, early Day-0 differences may reflect combined effects of clotting time, whole-blood exposure, and early tube interactions rather than gel adsorption alone. Fourth, we did not assess adsorption kinetics, free drug fractions, or direct drug–gel/metabolite–gel interactions, so mechanistic conclusions cannot be made. Finally, this observational study used residual clinical specimens, limiting control over patient-specific pharmacokinetic variables and pre-analytical handling conditions. These limitations indicate that our findings should be interpreted as technical observations under routine laboratory conditions, not as mechanistic conclusions.

## 5 Conclusion

Under the tested conditions, most analytes showed no clinically relevant concentration loss in the evaluated serum tube systems during 7 days of refrigerated storage. Clozapine showed a measurable and statistically significant decrease in separation-gel tubes but remained stable in non-gel tubes, suggesting an additional gel-associated contribution that may affect TDM interpretation when concentrations are close to clinical decision thresholds. Aripiprazole showed an early Day 0 tube-related difference without further storage-dependent loss. Because only one tube brand and gel formulation were evaluated and direct mechanistic experiments were not performed, these findings should be interpreted within the tested pre-analytical conditions. For clozapine TDM, non-gel serum tubes or prompt serum separation from gel tubes should be considered when analysis is delayed.

## Data Availability

The original contributions presented in the study are included in the article/supplementary material. Further inquiries can be directed to the corresponding author/s.
